# Manipulating Oxyphosphate of Zinc as a Filling-Material

**Published:** 1893-11

**Authors:** H. G. Register


ARTICLE IV.

### MANIPULATING OXYPHOSPHATE OF ZINC AS A FILLING-MATERIAL.

BY H. G. REGISTER.

In manipulating the oxyphosphate of zinc there is often a great deal of chemical action manifested in the form of the evolution of heat at the time of crystallization or setting. Under such circumstances the cement sets very rapidly, so that it becomes almost impossible to introduce it into the cavity and useless in the setting of crowns. The rapidity of setting and the intense heat produced are no doubt closely related as cause and effect. Where this condition is controlled the setting of the cement takes place more slowly, and if time is allowed for the perfect chemical combination of the acid and the powder a much denser and more satisfactory resulting cement is obtained. My experiments and observations lead me to believe that there is an exact chemical relationship between the quantities of ingredients which go to make up the final cement, which, if accurately observed, will produce the most satisfactory results; and variations from these exact relations are the principal causes which produce unsatisfactory variations in the quality of the cement. To secure the right quantitative conditions between powder and liquid, and to control the crystallizing process, the preparation is mixed upon a large bottle containing ice-water. The larger the volume of cold, the more manageable the result as to the time of crystallization, cold undoubtedly retarding the process.

In summer-time much more satisfactory results are gotten by using ice contained in a wide-mouthed jar (a fruit-jar will answer), and after filling with ice pouring in ice-water, which fills up the interstics and forces the ice against the glass. Using this as a base for the working of cement on warm days, when under ordinary circumstances it would set so rapidly as to be worthless, will give all the time desired to do the work leisurely and perfectly. In very hot and dry weather, if the retarding action of the cold is once discontinued, the setting of the cement is extremely rapid. I have noticed that where I have used the cement which under ordinary circumstances would set so rapidly as to be practically valueless, its setting could be perfectly controlled by the use of the cold mixing base. In manipulating the quickly setting cement in this manner, I have frequently noticed the following interesting phenomenon: I have incorporated powder with the liquid until it has become as tough and thick as putty, or until it would take up no more powder on the slab, but upon removing it from the cold base and manipulating it between the fingers it would return to an extremely plastic and sticky condition, previous to again becoming hard through the process of chemical combination or crystallization of its combining parts. In crown-setting, it is my habit to place the crown upon the cold base, upon which I at the same time mix the cement, in order that they may both be at the same temperature. I have also noticed that when a jet of heated air is directed on a filling of oxyphosphate which is undergoing the process of crystallization, the resulting mass becomes friable, with a tendency to granulate, owing to the increased rapidity brought about by the high temperature. It would seem better, therefore, to use a current of cold air or that of ordinary room temperature upon such fillings rather than a hot blast.

Thus prepared, and manipulated with platinum-pointed instruments, to which neither it nor gutta-percha will stick, it will give results which I believe to be the best that can be gotten with any of the zinc cements.—*Dental Cosmos.*

